# Cognitive Aspects of Comb-Building in the Honeybee?

**DOI:** 10.3389/fpsyg.2018.00900

**Published:** 2018-06-05

**Authors:** Vincent Gallo, Lars Chittka

**Affiliations:** ^1^Department of Psychology, School of Biological and Chemical Sciences, Queen Mary University of London, London, United Kingdom; ^2^Wissenschaftskolleg zu Berlin, Institute for Advanced Study, Berlin, Germany

**Keywords:** behavior, cognition, consciousness, planning, prediction, prospective cognition, wax

## Abstract

The wax-made comb of the honeybee is a masterpiece of animal architecture. The highly regular, double-sided hexagonal structure is a near-optimal solution to storing food and housing larvae, economizing on building materials and space. Elaborate though they may seem, such animal constructions are often viewed as the result of ‘just instinct,’ governed by inflexible, pre-programmed, innate behavior routines. An inspection of the literature on honeybee comb construction, however, reveals a different picture. Workers have to learn, at least in part, certain elements of the technique, and there is considerable flexibility in terms of how the shape of the comb and its gradual manufacture is tailored to the circumstances, especially the available space. Moreover, we explore the 2-century old and now largely forgotten work by François Huber, where glass screens were placed between an expanding comb construction and the intended target wall. Bees took corrective action before reaching the glass obstacle, and altered the ongoing construction so as to reach the nearest wooden wall. Though further experiments will be necessary, these results suggest a form of spatial planning skills. We discuss these findings in the context of what is now known about insect cognition, and ask if it is possible that the production of hexagonal wax combs is the result of behavioral heuristics where a complex structure emerges as the result of simple behavioral rules applied by each individual, or whether prospective cognition might be involved.

## Introduction

**FIGURE 1:**
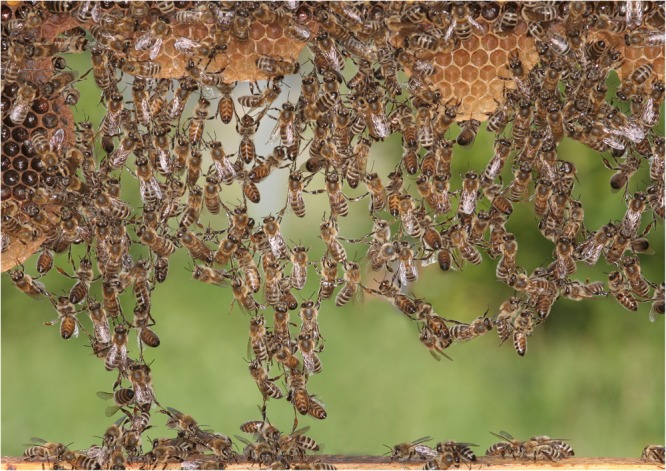
Construction of new comb in the honeybee *Apis mellifera*. The construction of hexagonal honeycombs requires the coordinated and cooperative activities of many dozens of individuals. Workers manufacture and manipulate wax into a highly regular hexagonal pattern (a mathematically close to perfect solution to honey and brood storage), and in the process have to evaluate the space available and the current state of construction, and process a diversity of communication signals from others, as well as proprioceptive input, for example to align the combs with gravity. These rich instinctual repertoires of many insects have often been thought to come at the expense of learning capacity. However, very few behavioral routines are fully hardwired and even comb construction skills have to be partially learnt by honeybees. Image by Helga Heilmann, with permission.

It has long been recognized that social insects have rich behavioral repertoires that orchestrate life in the colony, facilitate the elaborate construction of a communal home, secure a steady stream of appropriate food for their offspring, defend the colony and regulate its climate. This behavioral complexity has often been dismissed as ‘just instinct.’ Yet, recent discoveries in insect learning, memory and cognition have generated a profound change in the perception of the behavioral flexibility of several species. For example, bees learn from past experiences to improve motor skills (Mirwan et al., 2015; Abramson et al., 2016). Such operant learning is distinguished from cognitive operations, where, for example, bees are also able to combine multiple experiences (acquired in separate learning trials) to form simple rules and concepts (Giurfa et al., 2001; Avargues-Weber et al., 2012) and display counting-like abilities (Howard et al., 2018; Skorupski et al., 2018), and ants and bumblebees show simple forms of tool use (Loukola et al., 2017; Maák et al., 2017). Being capable of interval timing, bumblebees can predict future events (Boisvert and Sherry, 2006; Skorupski and Chittka, 2006). There is evidence that insects might at some level predict the outcomes of their own actions (Webb, 2004; Kim et al., 2015; Mischiati et al., 2015), or perceive a desirable outcome and then to explore possibilities to achieve this goal (Chittka, 2017; Menzel, 2017). In view of this, a re-evaluation of some behavioral routines traditionally thought to be entirely governed by instinct is in order (Bateson and Mameli, 2007). Even where behavior is partially instinctual, there can be multiple interactions with learnt behavior and cognition. Bird nest building, for example, was once thought to be wholly instinct-driven, but it is now apparent that many aspects of it can be experience-dependent (Walsh et al., 2013), and indeed the nesting instinct that requires the manipulation of elongated objects such as twigs in some birds can in turn facilitate cognitive behavior such as flexible tool use (Healy et al., 2008; Breen et al., 2016). In view of this, we here re-examine the learnt, and possibly cognitive, elements of what has been regarded by many as the pinnacle of animal instinctual behavior: the construction of the honeybee wax comb (**Figure 1**). Darwin referred to this as “the most wonderful of all known instincts" (Darwin, 1859, p. 235). Here we review the evidence that elements of comb construction need to be learned, and, exploring largely forgotten literature, how cognitive and planning skills might be involved.

## Optimality of the Comb Structure

**FIGURE 2:**
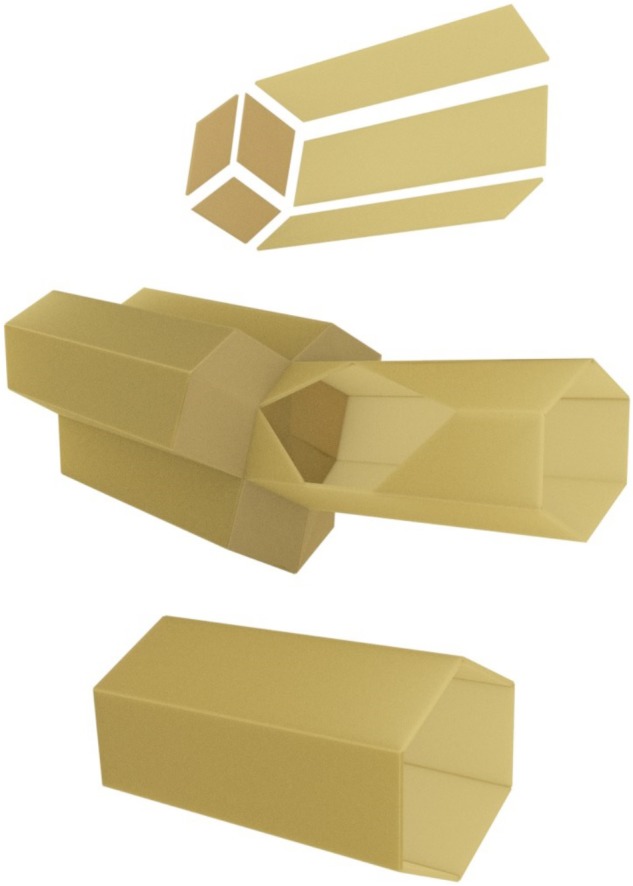
Schematic structure of hexagonal wax cells and double-sided honeycomb (computer graphic). Top: three walls and the three rhombi (forming the base of a cell) as discrete components. Central: a single cell, joined to three on the other side. The wall of the single cell is shown cutaway to reveal the cell base. Note that the cells slope slightly from the opening on each side, down toward the comb spine. The lower image shows a single drone cell, approximately 30% larger than cells built for worker larvae (as shown in the top panels).

**FIGURE 3:**
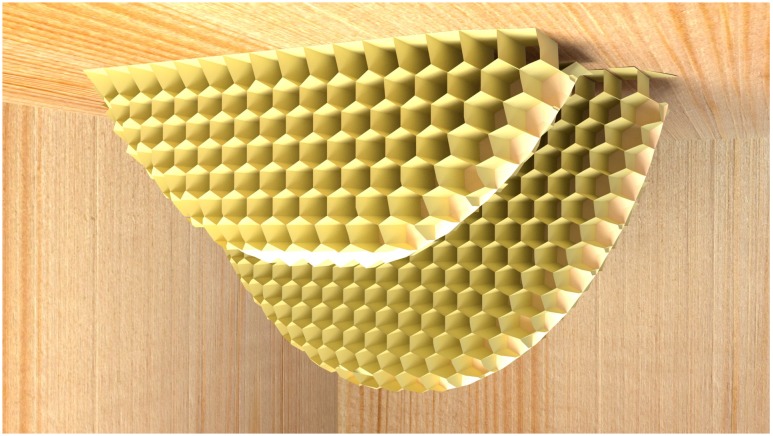
Comb construction of multiple parallel combs (computer graphic). The sketch shows how normal comb constructions of cavity-nesting honeybees where comb is begun attached to the top surface of the cavity, and then gradually extended downwards. Multiple combs will be grown, each roughly parallel and separated by a gap sufficient for the bees to work both. Note that the first line of cells (the “foundation”) is differently shaped to other cells. At the lower end of the construction, partially constructed cells come in a large variety of shapes, and individual workers can in principle continue from any partial construction.

The honeybee comb is, at first sight, a wonder of animal architecture. In all known species of honeybees, the structure is a double sided sheet of tessellated hexagonal cells where the base (common to both sides) is formed from three rhombi (**Figure 2**). Obviously, hexagonal cells are more suitable than the round cells used by, e.g., bumblebees, since the latter arrangement wastes a lot of space between cells. Square or triangular cells would have no gaps between cells, but since the larvae to be raised in the cells are neither square nor triangular in cross-section, space would be wasted inside the cells. Thus, hexagonal cells are intuitively suitable, and in fact some species of wasps build them too, albeit of “paper” (chewed wood) rather than wax. But no species of bee except honeybees also builds double-sided hexagonal combs — another notable strategy to save space and material. The bottom of each hexagonal cell has the shape of a pyramid (again a more efficient solution than a square bottom), and the two sides of the comb interface perfectly with one another through these pyramid-shaped bases of the cells. Unlike the combs of some stingless bees, the honeybee comb has to be vertical so that honey can be stored on both sides without dripping out, and the cells of the comb are tipped slightly downward from the opening to the base (**Figure 2**). In cavity-nesting species (*Apis mellifera* and *A. cerana*), multiple combs are built in parallel, leaving just enough space for workers to move about freely (**Figure 3**). This is despite the fact that cavities in which these species of honeybees nest naturally (e.g., hollow trees) are highly irregular in shape (not like the cuboid boxes beekeepers supply them with). Beyond the intuitive arguments in favor of a double sided hexagonal structure, it has been pointed out that the structure is in fact a mathematically optimal, or close to optimal, solution to economizing on building material while maximizing storage space (Kepler, 1611; Huber, 1814/1926; (Langstroth, 1853). Huber (1814/1926, p. 106) reported that the rhombus angles could beneficially be altered by modifying angles by 10 min. Analysis of the geometry of tessellated polyhedrons (Tóth, 1964) showed that the most economical cell construction (volume per wall area) comprised a hexagonal cell with a base formed from two squares and two hexagons. However, the saving would be less than 0.35%, at the expense of greater complexity of construction. By the use of self-aligning soap bubbles (Weaire and Phelan, 1994) it was shown that at a certain wall thickness, the ideal solution would switch from the optimal arrangement proposed by Tóth to that favored by the bees. We can thus infer that the structure is indeed very close to the theoretical optimum.

## Can Wax Comb Construction Be Explained by Simple Algorithms?

The repetitive structure of the comb seems like the perfect result of some robotic, hard-wired behavior routine — a kind of assembly line job of building the same structure over and over. It is tempting to assume some simple algorithm that might explain the shaping of the comb structure. For example, Pirk et al. (2004) proposed that each cell of the comb was a simple structure constructed from a curved wall, cylindrical tube, without facets or edges. The claim was that the temperature and fluid properties of the wax itself at the elevated temperature, present during comb construction, would, without further intervention by the bees, reform into the seemingly more complex hexagon by liquid equilibrium. This would be a process in which straight surfaces sometimes form in the same way as adjacent soap bubbles. However, thermal imaging technology (Bauer and Bienefeld, 2013) showed that the wax never achieved a temperature sufficient for reformation to occur. The precise geometry is not formed as a natural consequence of the material and temperature, but rather must be actively constructed by the bees, in the same way as wasps (even individual wasp queens) can fashion a hexagonal comb from plant material (Karsai and Pénzes, 2000). The search for parsimonious explanations in animal behavior, however attractive they may be, can sometimes lead in the wrong direction.

In fact, theoreticians sometimes overlook empirical work at odds with their “simple” explanations. While it is often possible to generate a similar outcome as that found in nature by means of modeling or engineering, such exercises can be reminiscent of inspecting a sophisticated piece of medieval embroidery, taking a photo of it, and saying “There! The photo has the same pattern! This means that we now understand how the embroidery pattern was generated.” Clearly, even if the result looks similar, we have not understood the technique by which the original was manufactured. This is a fundamental complication with many modeling approaches that try to explore how complex behaviors or constructions might result from “simple rules,” including existing ones for comb construction in paper wasps (Karsai and Pénzes, 1998; see Walsh et al., 2013 for an exploration in bird nest building). Any useful model of comb construction would have to take into account, at the very minimum, how slivers of building material are manipulated by an insect’s six legs and its mandibles, using its antennae and other sensors to assess where the construction needs to be amended and how, and processing information from other individuals, to ensure that efforts of multiple individuals complement each other to ensure that comb is built efficiently and ideally free of errors (departures from the ideal structure). It would have to consider the mechanistic or algorithmic process whereby a cell could be built by a programmed sequence of steps to masticate, deposit, press, sculpt, remove and replace material so as to form the faces, edges and thus the elemental cell.

The algorithm could be extended with repetition statements to form a regular sequence of cells against a flat horizontal surface, and further algorithm statements would be required to add the partial cells necessary to build horizontal cell layers against a sloped surface. Yet the task undertaken by comb-building insects goes further and copes with an irregular surface including fractures, cavities, and protrusions as is evident from their inhabitation of cavities within trees and rocks and by open nesting species of honeybees (such as *A. florea* or *A. dorsata*) that build externally on tree branches and/or rock. Not only must the builders overcome irregularities rooted in the shape of the support or cavity but also to notice and overcome errors introduced by themselves or other bees. This is not to say that elements of the social endeavor of comb construction cannot ultimately be explained by stereotyped behavior routines (and possibly in part relatively simple ones), but a model that does not incorporate these natural challenges of comb construction will oversimplify the problem, and generate an illusion of simplicity where there is none.

## Flexibility in How Individuals Build the Comb

Understanding the behavioral challenges of comb construction requires observation of individual and collective activities of bees engaging in small scale repetitive tasks, executed by many individuals, which collectively can lead to a multi-purpose structure to the benefit of the colony. The dexterity that is required for a six-legged animal to manufacture a repetitive structure with such regularity and precision is remarkable. In his classic work over two centuries ago, Huber (1814/1926) described the many variations that exist in the comb structure: for example, as bees build their comb in the typical manner from the top and working downwards, the first row of cells differs from subsequent ones since it functions as a foundation. One might suspect that worker bees use their own body as a sort of template to arrive at the correct dimensions of each comb cell — but this is certainly only part of the story, since the width of cells destined for drones is 30% larger (yet they are also built by workers). There are multiple other modifications of wax structure, e.g., for the wholly differently shaped cradles for queens, or the entombments for intruders such as mice that have strayed into a colony and are killed by bees. Huber describes in detail how comb construction is initiated by a single worker on the top of the hive, and how multiple individual workers sequentially contribute to the construction of each cell. He also describes the ability of honeybees to shape flat surfaces and angular connections, observing how bees form the rhombic bases by first sculpting the base from a “block” made from balls of wax, softened by a process of chewing and moistening. The beginning shape is subsequently enlarged by the addition of further balls of wax to form the cell walls and edges. The sculpting process, involving removal of surplus material, was described as being undertaken by a number of individuals, both successively and simultaneously, working on diverse sections. Different workers continue cells where others have left off (and do so correctly no matter the previous state of the cell), and inspect one another’s constructions to amend them where necessary. Huber (1814/1926, p. 129) noted several bees working on a small area of comb, one of which placed some wax in a misaligned location. An observant co-worker was seen relocating the wax better aligned to the current construction. These examples of adaptive behavior are of a small scale, correcting details of a scale less than that of a cell. Cell-scale adaptation of the construction method was also evident when a mixed species colony (*A. mellifera* and *A. cerana*) built comb over foundation ideal for one or other species (Yang et al., 2010). The mismatch between the natural cell size and that suggested by the foundation required adaptive modification of the bees’ natural construction habit.

Longer range flexible behavior can be seen where two or more festoons (hanging groups of comb forming bees) commence simultaneous construction of comb which, when enlarged, were sufficiently aligned so as to unite into a single blade. To create the connection between the two constructions, pentagons or heptagons are constructed (Hepburn and Whiffler, 1991). In that case the adaptation extends over several cells as to form a junction between misaligned combs. In another example of the bees’ flexibility, a hive of bees once traveled on board the Space Shuttle Challenger, 2 years before its doomed final mission in 1986. The honeybees spent an entire week in zero gravity. Not only did they learn to fly under such conditions, but they built honeycomb with cells of normal dimensions. The only difference (compared to honeycomb built on Earth) was that the cells of honeycomb were not consistently angled downward — perhaps unsurprisingly, since there is no obvious ‘down’ in zero gravity conditions for a honeybee (Vandenberg et al., 1985). But importantly, the geometry of the combs was correct — several combs had the usual straight and flat structure, and were built roughly in parallel, in the complete absence of gravity.

In conclusion, detailed observations of the comb building process reveal that multiple behavioral routines might be at work and are subtly tailored to need. Many of them might still be governed by hard-wired, innate routines, but they seem far from simple, given the versatility and flexibility observed. In what follows, we examine the literature indicating that learning and cognition are also involved.

## Possible Elements of Learning in Comb Construction

In natural bee comb constructions, there are a variety of subtly different ways in which wax comb is structured (especially with respect to how the two sides of comb are interfaced). The way in which young workers build comb is affected by the structure of the comb they were raised in, and were allowed to sample for some time after emergence (von Oelsen and Rademacher, 1979). In a similar vein, Martin Lindauer discovered that, after swarming and relocating to new home, the combs in the new home would typically have the same angle to the Earth’s magnetic field as the natal nest, indicating that bees had memorized this angle and then replicated it in the new construction (Seeley et al., 2002). While these observations are indicative of an importance of learning in comb construction, it might also be possible that there are genetic effects that determine comb structure and orientation.

The classic approach to investigate whether behavioral routines present in adults are innate or learnt is to experiment on individuals reared in isolation, under conditions where they have no exposure to the behavior in question, or to the desired outcome of the behavior. For questions of comb construction, such experiments were first performed with orphaned *Poliste*s wasps, and it was observed that the comb geometry in such wasps departed from the usual radial symmetry (Rau, 1929). von Oelsen and Rademacher (1979) reared honeybee larvae, removed from their natal comb, in circular plastic cells. Such individuals later managed to build hexagonal cells, but with highly variable cell dimensions. In addition, peculiar modifications were apparent in the comb structure beyond that of the single cell. Bees raised without having experienced normal honeycomb built irregular bases and unconventional cell arrangements (rotated or floral configuration) while juveniles that had been allowed access to conventional comb and/or experienced workers built conventional comb. As with many other behaviors, e.g., bird song, innate predispositions only provide a rough template for acceptable behavior in the adult — the details need to be learned (Thorpe, 1973; Mets and Brainard, 2018).

Learning can also be apparent in insects’ ability to repair experimentally damaged comb. Working with *Polistes* wasps, Downing and Jeanne (1990) observed that when holes were made in the existing comb structure, the time to repair them decreased with repeated exposure, and individual wasps improved their repair technique with experience. For an example of repairing accidental damage in honeybees, see subsequent section.

## Possible Cognitive Aspects and “Planning” in Comb Construction

Comb building capabilities, and the degree of adaptability and individual or collective cognition necessary to achieve the outcome, can be investigated by disrupting or interrupting the normal process. Remarkably visionary experiments on the flexibility of honeybee comb building were described in Huber, (1814/1926) work. Under natural conditions, the comb constructions of cavity-nesting honeybees are attached to the top surface of the cavity and then gradually extended downwards (**Figures 1, 3**). These bees naturally nest in hollow trees, and therefore typically attach comb to wooden surfaces. To observe a bee colony’s inner workings over extended periods, Huber replaced various walls of the hive with glass, and found that when given the choice, bees rejected slippery glass surfaces as starting attachment points for honeycomb construction. When Huber used a glass lid rather than wood for the roof of the hive box, he found that the bees built the honeycomb from bottom to top. The entire building process was thus inverted, with the comb base adhering to the lower horizontal surface, and bees were building cells from the lower side upwards. The upper edge of the comb was curved as it was grown (in the same way as the tip of normal, downward-growing comb is curved). Note that this is far from trivial: the challenge of having a glass ceiling is one that no bee colony would ever have encountered in its evolutionary history. In addition, since the motor routines linked to comb construction are typically aligned with gravity (in the downward direction), bees would have to reverse the contingencies between gravity and the appropriate motor routines in order to build honeycomb of the correct geometry.

Later experiments were designed (Huber, 1814/1926, p. 157) to further coerce the bees into building laterally, achieved by providing a wooden wall but glass roof and floor. Again, the bees were able to adjust their building methods to cope. In that case, they started at one of the side walls and extended the comb laterally across the cavity. It is useful to compare this flexibility with that displayed by other animals whose nest construction has been studied in some detail. Some species of African weaver birds build elaborate all-round enclosed nests that are woven together from grass blades and suspended from tree branches (Walsh et al., 2013). A comparable experiment to that of Huber’s would be to prevent weaver birds from access to branches from which to hang their nest; would they be able to build a nest “bottom–up” on a stilt attached to the ground, or one that is at least built directly on the ground? Perhaps they could, but if you further prevent them from using the ground beneath from building their construction, could they suspend a nest between two vertical poles? If they did, you would rightfully conclude that the weaver birds’ building behavior is not tightly ruled by hard-wired behavior routines, but that they instead have an awareness of the desirable outcome of their activities, and subjugate their (perhaps partially innate) nest building activities to this outcome. The same interpretation thus should be considered for Huber’s findings on bees’ building activities.

But Huber’s next experiment is perhaps the most remarkable in that it is reminiscent of present day attempts to study animal intelligence by way of their responses to transparent obstacles. In these more recent experiments, a transparent screen is placed between the animal and its target (typically food), and the animal’s learning speed in suppressing direct movements to the target (and instead to circumvent the transparent material) is measured (MacLean et al., 2014). This paradigm has been used to compare self-control (as an indicator of cognitive ability) between vertebrate species, though this approach is not without complications (van Horik et al., 2018).

**FIGURE 4:**
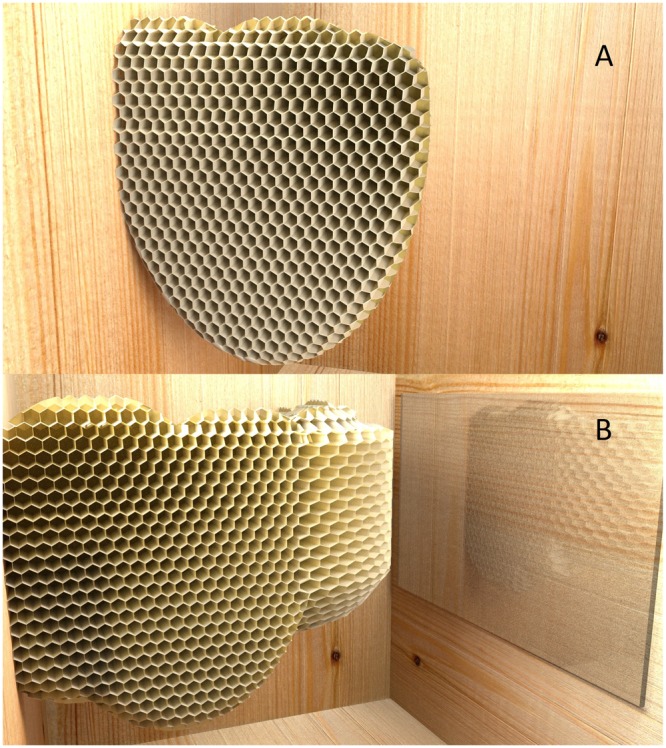
An experiment by Swiss entomologist Huber (1814/1926) to probe the flexibility of the honeybees in comb construction in the face of unusual challenges (computer graphic). Huber had noticed that bees avoid, when possible, to attach the comb construction to glass walls of observation hives. **(A)** When bees were faced with the hive that had a glass ceiling and floor, they would begin their construction on one of the side walls. **(B)** When the bees had not yet reached the target wall, a glass screen was placed over that wall. Rather than continuing the construction into the same direction, the bees introduce a curve into the construction by building cells with expanding sizes on the outside of the curve, and cells with reduced orifices on the inside. Continued construction of the comb in the revised direction results in adhesion to a more suitable target area for attachment.

In Huber’s experiment, it was not the individual animal’s path that had to be adjusted to the appearance of a transparent obstacle, but the trajectory of the growing wax comb. The target in this case was not a food item, but to attach the opposite end of the comb to a suitable vertical surface. After lateral comb construction had begun, Huber placed additional sections of glass to cover the wall toward which the construction was aimed. He anticipated that perhaps once the bees had reached the glass, they would make some sort of special efforts to attach the comb to this suboptimal and slippery surface. But they did something else altogether: apparently noticing that their intended target surface had been rendered suboptimal, the bees took corrective action and turned the construction of their comb by 90° — before their construction had reached the target wall (**Figure 4**). Though these experiments are not identical to those designed to test vertebrates’ responses, it is noteworthy that no vertebrate displays a spontaneous avoidance of glass obstacles when they are first placed in front of their target; all have to learn from the experience of “bumping into” the obstacles (MacLean et al., 2014).

Huber reported that he repeated this experiment in multiple ways, sometimes moving the glass target into the projected path of their comb building activity several times, and bees would change the direction of their construction again and again. Huber observed that bees had to change the dimensions of the hexagonal wax cells around the kink; the comb cells on the outside surface were 2–3 times wider than on the inside.

We can dismiss the possibility that bees have an innate response to the glass obstacle between their comb construction site and the intended target wall, since such a challenge has never been encountered by bees in their evolutionary history. Simple learning and memory processes cannot easily explain how an animal copes with wholly novel challenges either, though a non-cognitive explanation of the bees’ behavior might begin something like this: since Huber had previously introduced a glass ceiling and roof to the hive box (to force the bees building a laterally expanding comb attached to one of the side walls), the bees had gained experience with glass surfaces and their suboptimal properties in terms of attaching wax. When the new glass screen was inserted on the wall opposite the one where the comb construction had been started, the bees at the front of the construction saw the transparent obstacle whose visual appearance they had previously associated with poor adhesiveness (since Huber’s observation hives had a glass lid, it was not dark as in normal beehives, so unlike natural conditions, bees could theoretically have used vision to guide their comb construction). As a result, the bees would have looked around for more suitable target locations to which to attach the comb, and subsequently altered the direction of the expanding comb. In that view, the alteration of the comb construction would be little more than a form of aversive conditioning, where bees simply avoided the glass obstacle that had been placed in their way. Perhaps the construction troupe acted like a swarm of flying birds that is suddenly faced with an obstacle in their path, and took evasive action around the looming stimulus?

There are complications with this “simple” explanation. Even if bees had previously learned to link the visual appearance of glass with poor wax adhesion, it is unclear whether vision would have helped with the solving of the task. Unlike the transparent glass ceiling, the altered target wall is a sheet of glass with a wooden wall behind, and the fact that there was now glass in front of the wood could only have been deduced from subtle mirroring effects (see **Figure 4**). It is uncertain whether bees would be able to see such effects, especially given their poor visuospatial resolution (Spaethe and Chittka, 2003). An alternative that remains to be explored is whether “scouts” assessed the suitability of the target wall by tactile sensing, and then returned from this wall to the construction site, reporting in some way that this wall was no longer suitable. But whether or not the suitability of the target wall is assessed by visual or tactile means, the fact remains that this assessment was done at a distance, before the target wall had been reached — i.e., the bees must have found a way to extrapolate from the current direction of the comb construction to even assess the suitability of the surface to which it might be attached in future, when the comb construction would have advanced further. From Huber’s descriptions of the geometry of the experiment, we conclude that the distance at which he introduced the glass screen must have been a minimum of 5 cm (but likely multiple times this) from the tip of the wax construction site. From empirical information about the natural speed of comb construction, it would have taken at least half a day to bridge the remaining distance (Freudenstein, 1961; Hepburn et al., 2014, p. 26). The analogy with the flying bird swarm (in the previous paragraph) thus does not hold in several respects: the wax-constructing bees are not “forward facing” (depending on the current building activity, they might have their heads stuck in partially constructed cells, and of those on the outside of the construction, only a minority will face the direction of the obstacle. Moreover, because of the slow growth of the comb construction, there is no looming stimulus (an obstacle whose apparent size rapidly expands as the subject approaches) – thus there is no simple way to predict the location of contact with the target zone from rapid sensory stimulus change. In addition to predicting the target zone that the construction would have reached many hours or indeed days later, there must have been a process (either visual or tactile) to identify *more* suitable areas to attach the comb in future, before the direction of building was altered.

At the very least, the following questions must at some level be answered by the comb construction troupe: if we continue building in the current direction, which area of the opposite wall will we reach? Is the surface of this area suitable? If it is not, then what are suitable alternative target areas? After identifying a suitable target area, what is a suitable alteration of current comb building direction to reach that target area in a straight line? A possible cognitive explanation for the bees’ collective correction of comb geometry is that there was an appreciation of the possible (suboptimal) outcome of the construction, were it continued in the initial direction, though this interpretation should be substantiated with further experimentation.

Finally, there remains the question of how the many bees engaged in the construction site agree on changing the direction of the comb. The two basic options are to angle the comb construction to the left or to the right, but more subtle decisions also need to be made: i.e., should the new section of comb be perpendicular to the existing construction, perpendicular to the new target wall, or some oblique angle to either. Whatever the chosen direction, all bees would have to agree; otherwise a lacerated construction would result. That bees (and other social insects) are able to form a consensus among multiple possible options is well-known from the context of searching for, and agreeing on, a new nesting site (Dornhaus and Franks, 2006; Seeley, 2010). However, the heuristics used in this search are related to challenges that have been faced by these insects under natural conditions for millions of years and are therefore shaped by natural selection. Huber’s glass wall experiments faced comb-constructing honeybees with a task unprecedented in their history as individuals and as a species. Nonetheless, as a group, they were able to form a consensus for how to best address the challenge. There is, however, a need to replicate these experiments with more detailed recordings of which individuals do what, in the process of assessing suitable target locations for the comb, as well as during the decision making of how to alter the construction.

Natural behavior that appeared to anticipate a need that has yet to arise was also reported by Huber (1814/1926, p. 175). During winter, foraging for flowers, brood rearing, and indeed comb construction is halted, and bees will minimize any activity to ensure that their storage lasts until spring. On one occasion, Huber observed that one of several combs broke off the ceiling of the hive. Not only did bees become active to fortify the dislodged comb with a number of pillars and cross-beams made from wax, but they subsequently also reinforced the attachment zones of all the other combs on the glass ceiling, to ensure that a similar disaster won’t happen again. Wrote Huber: “I may restrain myself from reflections and commentaries, but I acknowledge that I could not suppress a sentiment of admiration for an action in which the brightest foresight was displayed.” If such anecdotal reports could be verified with multiple replicates under experimental conditions, these results might indeed be examples of prospective cognition or foresight (Clayton et al., 2008; Crystal and Wilson, 2015).

One might counter that the precautionary repairs induced by the mid-winter accidental damage, as well as the responses of bees to Huber’s experimental manipulations, might not necessarily be based on foresight – that instead that they might be based on a very large number of hard-wired routines, all triggered by a certain stimulus configuration. This is possible, but one should also consider whether postulating that such a repertoire that includes appropriate responses to every tested experimental manipulation is any more parsimonious than claiming that they do require a form of planning. The challenge would be to explain how such preventive behavioral measures can occur as a result of natural selection. This may be just plausible in the case of preventive midwinter comb fortifications, but it will be very difficult to argue how the anticipatory responses prompted by Huber’s experimental manipulations should occur as a result of evolutionary processes — when evolution is very unlikely to have ever presented the kinds of circumstances that Huber faced the bees with. In searching for parsimonious explanations, it is not adequate to use intuitive arguments about which path to the same behavior *looks* less or more complicated by casual inspection (Chittka et al., 2012). For an evolutionary scenario, one would have to consider which neural circuitry tweaks are necessary for an animal to turn from one that constructs honeycomb by simple robotic principles to one that masters all the unusual challenges above, the mutations that would be required, the environmental conditions that would favor each step. Could it be that a cognitive scenario – where bees have an appreciation of the desired outcome of the comb construction, where behavioral routines are employed relatively flexibly toward reaching the desired goal – could actually be a mechanistically simpler explanation than one that includes a large variety of fixed-action patterns and cognitive tools, including for scenarios that bees won’t typically encounter under natural conditions? It is important here to realize that the neuron numbers and circuitries required for agents that can foresee the outcomes of their own actions are certainly not prohibitively large even for insect brains (Shanahan, 2006), and indeed such an ability might have arisen relatively early in evolution as a powerful instrument to solve common but also more unforeseeable challenges in animals’ lives (Bronfman et al., 2016).

## Conclusion

A traditional idea is that animals have an easily classifiable repertoire of motor routines (in the same way as a Swiss army knife has a limited number of tools with defined functions). For example, perhaps you were taught in school that horses have three gaits (walk, trot, and gallop) and humans two (walk and run). While indeed there may be certain default classes of locomotion in any species, it is clear that humans are capable of an infinity of others — you can crawl, walk on all fours, jump on one leg, walk on crutches, etc. You can easily adapt your locomotion to your current need, your spatial environment, any form of injury, etc. In the same vein, bees by default build hexagonal cells of two dimensions (smaller ones for workers, larger ones for drones), but depending on need, they can also build pentagonal or heptagonal cells, cells that are wider or smaller near the orifice than they are at the base, or use wax for building barriers at the hive entrance to keep out intruders, etc. Huber (1814/1926, p. 178). The historic distinction between behavior being governed by either instinct or learning/cognition is no longer tenable; instead there are interactions at multiple levels and indeed certain instinctual routines that come with an animal’s ecological niche will in turn favor certain forms of cognition (Bateson and Mameli, 2007; Audet and Lefebvre, 2017; Robinson and Barron, 2017). For example, all healthy humans have an innate predisposition for language (an ‘instinct’) (Pinker, 1954/1994), but having the language instinct facilitates almost all cognitive abilities that we pride ourselves in, including the capacity for cultural evolution, or theory of mind (knowing what others know) (Sacks, 1989). In the same vein, the instinct that determines bees’ dietary specialization as consumers of floral nectar and pollen (as opposed to being, e.g., carnivores or parasites) in turn requires them to learn about floral features. We have here dissected a behavior that has been traditionally thought to be wholly governed by instinct. The comb construction abilities demonstrated by honeybees extend beyond a simple algorithm of applying wax to a set pattern; rather, adaptability and error recovery are evident. The insects have a number of perhaps basic, partially hard-wired routines to manufacture the elemental structure of the hexagonal cell (von Oelsen and Rademacher, 1979), but also have the capability to adapt the basic method in order to overcome errors or incompatibilities, to observe and remedy perturbations, to use parts of an elemental cell to correct surface irregularities or to join incompatible sections and, where continued growth would be inadvisable, to take corrective action (Huber, 1814/1926). Huber’s classic work suggests that honeybees, rather than building wax comb in the way a robot might, may possess a “master plan” of the desired outcome, and can tailor their efforts to achieve this goal. Such an interpretation is consistent with recent explorations of intentionality or consciousness-like phenomena in bees (Barron and Klein, 2016; Menzel, 2017; Perry et al., 2017).

## Author Contributions

VG and LC contributed equally to the conception and writing of the content for the review.

## Conflict of Interest Statement

The authors declare that the research was conducted in the absence of any commercial or financial relationships that could be construed as a potential conflict of interest.
